# Structural influence of gene networks on their inference: analysis of C3NET

**DOI:** 10.1186/1745-6150-6-31

**Published:** 2011-06-22

**Authors:** Gökmen Altay, Frank Emmert-Streib

**Affiliations:** 1 Computational Biology and Machine Learning Lab, Center for Cancer Research and Cell Biology, School of Medicine, Dentistry and Biomedical Sciences, Queen's University Belfast, 97 Lisburn Road, Belfast, BT9 7BL, UK; 2 Cambridge Research Institute, Department of Oncology, University of Cambridge, CB2 0RE, Cambridge, UK

## Abstract

**Background:**

The availability of large-scale high-throughput data possesses considerable challenges toward their functional analysis. For this reason gene network inference methods gained considerable interest. However, our current knowledge, especially about the influence of the structure of a gene network on its inference, is limited.

**Results:**

In this paper we present a comprehensive investigation of the structural influence of gene networks on the inferential characteristics of C3NET - a recently introduced gene network inference algorithm. We employ local as well as global performance metrics in combination with an ensemble approach. The results from our numerical study for various biological and synthetic network structures and simulation conditions, also comparing C3NET with other inference algorithms, lead a multitude of theoretical and practical insights into the working behavior of C3NET. In addition, in order to facilitate the practical usage of C3NET we provide an user-friendly R package, called *c3net*, and describe its functionality. It is available from https://r-forge.r-project.org/projects/c3net and from the CRAN package repository.

**Conclusions:**

The availability of gene network inference algorithms with known inferential properties opens a new era of large-scale screening experiments that could be equally beneficial for basic biological and biomedical research with auspicious prospects. The availability of our easy to use software package *c3net *may contribute to the popularization of such methods.

**Reviewers:**

This article was reviewed by Lev Klebanov, Joel Bader and Yuriy Gusev.

## Background

A systematic understanding of biological and biomedical problems can only be achieved if the working mechanisms of molecules in cells of an organism are sufficiently understood. Since the postulation of the *one gene-one enzyme hypothesis *in the early 1940 s by BEADLE and TATUM[1], which sparked molecular biological studies for decades, the current focus of research has shifted toward systems properties of interacting genes [2-7]. With the advent of high-throughput data, we are nowadays in a position to study the behavior of such systems quantitatively. For instance, microarray experiments provide powerful data sets containing a wealth of information about the expression of genes that can be exploited by statistical analysis methods in order to interrogate data systematically [8-14].

One aspect in the context of the analysis of microarray data that gained recently widespread interest is the inference of causal interactions among hundreds or thousands of genes [15-22]. Here by *causal *we mean the direct interactions among genes that correspond to experimentally verifiable biochemical interactions. It has been recognized that gene regulatory network inference (GRNI) algorithms are an important means to obtain genome-scale causal interaction networks which seem more amenable for a functional interpretation than other types of data representations [2]. Among the best GRNI methods are information theory based approaches [23-28]. A special subclass of such approaches are inference methods based on estimates of mutual information (MI) values [23,29,30]. In contrast to, e.g., the Pearson correlation coefficient, MI values are capable of detecting linear and non-linear effects among gene pairs and, hence, may be more appropriate in a genome context [31,32]. Recently, a new GRNI algorithm, C3NET [33], has been introduced. C3NET, which is also MI-based, has been compared with other GRNI algorithms [33], including ARACNE, MRNET, CLR and Relevance Network (RN) [27,30,34,35], by using F-scores as performance metric.

The major purpose of the present paper is to analysis the inferential characteristics of C3NET with respect to different network types of gene networks. Specifically, we will study the influence of various network structures, two biological as well as three synthetic ones, using global and local performance metrics. In our previous study [33], C3NET has been introduced and studied by using biological networks only. Also, these studies have been conducted focusing exclusively on global performance metrics in form of F-scores. Instead, in this paper we will use four different types of local-network based measures to assess the performance of C3NET. The utility of local network-based measures has been shown in [36,37]. Briefly, global measures like the F-score provide only partial insights into the intricate inferential assessment because they average over the entire network structure resulting in a global performance measure. However, there may be parts or subnetworks, e.g., motifs or modules, of the overall network that may be significantly better to infer than others. In oder to identify such substructures local network-based measures allow to *zoom in *these structural regions. In addition, we provide an introduction to the usage of *c3net*, a R implementation of C3NET.

Studying the influence of different network structures on the inferential characteristics of any inference algorithm is an important task for several reasons. First, our knowledge about the causal interactions of genes or gene products is still quite limited, especially for higher organisms like mouse or human. For this reason, we need to rely on simulation studies guiding the selection of GRNI algorithms that could be applied to novel data sets from expression experiments. Because of potentially unknown features of the regulatory networks, underlying these expression data, which may lead to deviations to other networks for which the GRNI algorithm has been tested, knowledge about the robustness of a GRNI algorithm is an essential property that needs to be taken into account when selecting a GRNI algorithm. Second, due to the fact that the robustness of a GRNI algorithm is directly connected to the study of different data sets, respectively their underlying network structures, this property cannot be studied by using one or two network types only. Instead, a sensible variety of network types needs to be considered from which simulated expression data can be generated on which the GRNI method is applied to. Regarding a more technical point, in addition to these studies we investigate the dependency of the inferential performance of C3NET on the MI threshold, or cut-off value, used to eliminate non-significant MI estimates.

This paper is organized as follows. In the next section we describe the methodology and our simulation set-up used for our numerical analysis. In addition, we provide a description of a R package we implemented providing the C3NET algorithm. In the results section we study the inferential behavior of C3NET for various network types, biological and synthetic networks, by using different performance measures. The paper ends with discussions and concluding remarks.

## Methods

**Figure 1:**
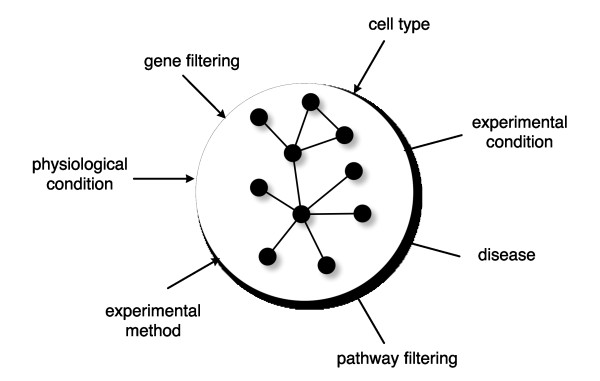
**Illustration of the general influence that various conditions have on the structure, the characteristics and the size of gene networks**.

One major objectives of this paper is to analyze the influence of different structures of gene networks on C3NET. Our rational for this is at least three fold. First, the knowledge of the *true *structure of transcription regulatory, metabolic or signaling networks is still at its infancy. For this reason it is difficult to select a *priori *a single synthetic network type which is best for testing an inference algorithm. This is even more true in the presence of a disease which may lead to the rewiring of smaller or larger portions of one or more of the above mentioned gene network types. Hence, in order to allow the application of an inference algorithm to data sets from biomedical or clinical studies, with potentially difficult to anticipate interaction patterns, a broad analysis is indispensable. Second, despite the fact that it is commonly acknowledged that the degree distribution of gene networks follows a power law, this property does not define the structure of a network uniquely. For instance, the *preferential attachment *algorithm [38] leads to such a scale-free degree distribution, however, does not generate a module structure [39]. This is in contrast to, e.g., the protein interaction networks of many species, possessing an intricate module structure. Unfortunately, there is currently no general agreement what network properties should be present in addition to the scale-freeness of the degrees, again, especially in the context of biomedical and clinical data. Third, if the underlying data come from microarray experiments it is common to filter genes according to certain criteria, resulting in a subset of genes. Also, there may be reasons to focus only on genes coming from a limited number of biological pathways, e.g. apoptosis or cell cycle. All these filtering procedures lead to a limited subset of genes whose underlying gene network may have a complex structure difficult to anticipate. Figure 1 gives a schematic overview of various influences that determine the structure, characteristics and size of gene networks. Some of these conditions may be controllable by the investigator whereas other are not.

### Brief overview of C3NET, RN, ARACNE and MRNET

**Figure 2:**
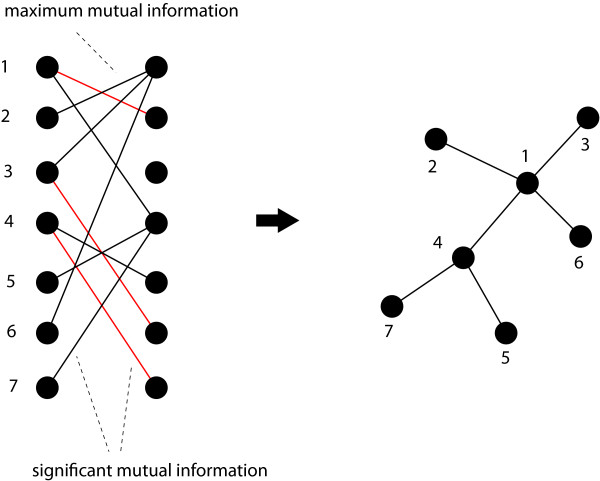
**Principle working mechanism of C3NET**. The edges shown in red and black correspond to significant edges. The edges in black correspond to the maximum mutual information valued edges for the gene on the left hand side.

In this section we provide a brief overview of the GRNI algorithm C3NET, introduced in [33]. Principally, C3NET consists of two main steps. The first step is for the elimination of nonsignificant edges, whereas the second step selects for each gene the edge among the remaining ones with maximum mutual information value. The first step is common to many GRNI algorithms, for instance Relevance Network (RN) [23], ARACNE [30], MRNET [27] and CLR [35]. This statistical inference step is essential to eliminate nonsignificant links, according to a chosen significance level *α*, between gene pairs. In the second step, the most significant link for each gene is selected. This link corresponds also to the highest MI value among the neighbor edges for each gene. This implies that the highest possible number of edges that can be inferred by C3NET is equal to the number of genes under consideration. This number can decrease for several reasons. For example, when two genes have the same edge with maximum MI value. In this case, the same edge would be chosen by both genes to be included in the network. However, if an edge is already present another inclusion does not lead to an additional edge. Another case corresponds to the situation when a gene does not have significant edges at all. In this case, apparently, no edge can be included in the network. Since C3NET employs MI values as test statistics among genes, there is no directional information that can be inferred thereof. Hence, the resulting network is undirected and unweighted. The principle working mechanism of C3NET is visualized in Figure 2. For a detailed explanation of C3NET, the reader is referred to [33].

**Figure 3:**
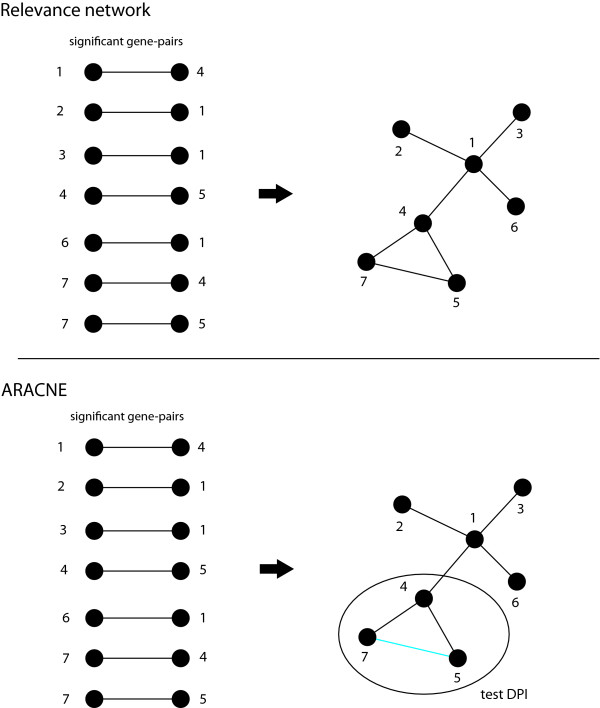
**Illustration of relevance network (top) and ARACNE (bottom)**. Each inference algorithm determines significant mutual information values. ARACNE applies in addition a second step, testing each gene-triple with the DPI.

The principle idea of RN [23] is to compute all mutual information (MI) values for all pairs of genes and declare mutual information values as significant if their corresponding value is larger than a given threshold *I*_0_. The first step of ARACNE [30] is similar to RN. In a second step ARACNE uses the *data processing inequality *(DPI) to eliminate the smallest mutual information value of gene-triplets if this value is below a certain threshold. The parameter that controlles this threshold has been called the tolerance parameter [30]. Both methods are visualized in Figure 3.

MRNET [27] is an iterative algorithm that identifies potential interaction partners of a target gene Y that maximize a scoring function.(1)(2)

When a gene, *X_j _*, is found with a score that maximizes Eqn. 1 and *s_j _*is above a threshold, *s*_0_, then this gene is added to the set S. The basic idea of MRNET is to find genes that are of maximal relevance (first term in Eqn. 2) for *Y *, but introduce a minimum redundancy (second term in Eqn. 2) with respect to the already found interaction partners in the set *S*.

### Implementation of C3NET: Usage of the R package

In order to make C3NET usable for biologists we implemented a R package called *c3net*. The software package c3net is available from the web site https://r-forge.r-project.org/projects/c3net and from the CRAN package repository.

To illustrate the principle working mechanism of the package we provide an example data set for which we discuss in the following its analysis. The data set and the true network are loaded in R by executing the *data(expdata) *and *data(truenet) *commands of c3net. Here, the variable for the data set is *expdata *and the variable for the true network is *truenet*. There is a compact function available in the *c3net *package with the same name, *c3net*, that takes the data set as input and outputs the inferred network. This is convenient for the user, because it hides the complexity of individual steps of C3NET, providing an all-in-one single command. The usage of the function and its default parameters are as follows: *c3net(dataset, alpha = 0.01, methodstep1=*"*cutoff", cutoff MI = 0, MTCmethod=*"*BH", itnum = 5, network = FALSE)*. Here *dataset *is the data set and *alpha *is a user defined significance level *α*. For the parameter *methodstep1 *one can assign three different options, {"*cutoff*", "*MTC*", "*justp*"}, in order to choose a procedure to eliminate nonsignificant edges. If *methodstep1 *= "*cutoff*" then its dependent parameter *cutoffMI *needs to be set to a numerical value that is used as cutoff value to eliminate nonsignificant MI value of edges in Step 1 of C3NET. If *methodstep1 *= "*MTC*" then a multiple testing correction (MTC) method is used in Step 1 of C3NET. In this case, a MTC method needs to be specified by the dependent parameter *MTCmethod *(e.g. *MTCmethod*= "BH"). Available options of different MTC methods are *"BH", "bonferroni", "BY", "hochberg", "holm", "hommel"*. Also, *itnum *needs to be set to specify the number of iterations to obtain a null distribution and *alpha *the statistical significance level. If *methodstep1 *= *"justp" *then only *alpha *and *itnum *need to be set and the elimination in Step 1 is done with respect to the p-values and the significance level *α *only.

In addition to providing the inference procedure of C3NET [33], the *c3net *package allows also a visualization of the inferred network by utilizing the *igraph *package [40]. This can be accomplished by setting the parameter *network *to *TRUE*. As an example one can execute *net *= *c3net(expdata, network = TRUE)*. Further, *c3net *provides a function to validate the performance of the inference called *checknet*. This evaluation can be obtained by executing *checknet(net, truenet)*. The *checknet *function results in the following six values: precision, F-score, recall, TP, FP and FN. For the provided example data set the function *checknet *gives precision = 0.96, F-score = 0.34, recall = 0.21, TP = 181, FP = 6, FN = 683. We would like to emphasize that the *c3net *package provides additional functions that allow to perform individual steps only instead of performing the entire inference step. This allows a flexible combination with components outside our package the user may want to use.

In order to learn the usage of *c3net *quickly we compiled a file with the name EXMAPLE.TXT containing examples that can be executed line-by-line demonstrating the functionality of c3net. Additional help for each function of *c3net *is available by using the *help *function for each command. Further, we produced a vignette file which can be found in the *inst *folder of *c3net *where all explanations and examples of the functions of *c3net *can be found in a PDF document.

### Simulation methodology

**Figure 4:**
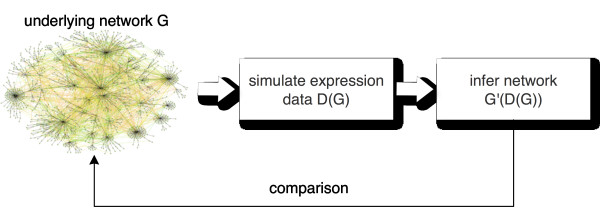
**Illustration of the dependencies of the data and, hence, of the inference algorithm on the underlying network structure**.

For our study we use two biological and three synthetic networks, each consisting of 100 genes. As synthetic networks we use a directed acyclic graph (DAG) [41], a scale-free (SF) network [38] and a random network (RND) [42]. To generate these networks we used GeNGe [43]. The real biological networks were obtained by randomly sampling a subnetwork of size 100 from the transcriptional regulatory network of *E. coli *[44,45] and of *yeast *[46] by using SynTReN [47]. Our overall simulation set-up is shown in Figure 4. It is important to emphasize that any network inference algorithm operates on data *D*, which are a function of the underlying network *G*. Hence, the inferred or estimated network *G' *is a function of *D*(*G*). For this reason, variation of the structural connectivity of a network has a crucial influence on the dynamic states of the network and, consequently, on the performance of an inference algorithm.

For each of the above networks, we generated simulated expression data, including biological noise, by using SynTReN [47]. The noise model used by SynTReN is a lognormal distribution. This model is supported by experimental findings [48]. We repeated this step 300 times, generating 300 different data sets for each network type by changing the kinetic parameters for each simulation at each step. This way the biological variability of a population of similar organisms is imitated. We used these data sets for an ensemble-based performance analysis. This allows to measure important network statistics of C3NET, both on the global and local network level, for each network. In total we generated 2100 gene expression data sets with steady-state values of varying sample sizes. These data sets were copula-transformed before applying the MI estimation algorithm [30]. For the estimation of MI values a nonparametric Gaussian estimator was used [26,49]. The DPI tolerance parameter of ARACNE, when used for comparison purposes, was chosen as 0.1 [30].

In the first step of C3NET, aiming at the elimination of nonsignificant edges, we used the optimal cut-off value, which is the threshold (*I*_0_) that maximizes the F-score for each data set with respect to the true underlying network structure [36,37]. We want to emphasize that also for all other methods studied we used their optimal threshold *I*_0_. The F-score is defined as *F *= 2*pr*/(*p *+ *r*), where *p *corresponds to the precision, *p *= *TP*/(*TP *+ *FP*), and *r *to the recall, *r *= *TP*/(*TP *+ *FN*). Here TP (true positives) is the number of correctly inferred edges, FP (false positives) the number of incorrectly inferred edges and FN (false negatives) the number of true edges that could not be inferred.

## Results

In the following sections we investigate the inferential characteristics of C3NET with respect to the structure of the underlying gene network by using two fundamentally different types of performance metrics. One type of metrics allows a global analysis of the inference algorithm only, the other permits a local one, allowing to *zoom in *building blocks of the network and their assessment.

### Global performance metric

In this section we investigate the global inference performance of C3NET for five network types and different sample sizes. We also compare it with two other GRNI methods, namely ARACNE and MRNET. Table 1 summarizes our results. From this table we see that, in general, C3NET gives higher F-score values than the other inference methods for the studied cases. Specifically, in three out of five cases the median (and mean) F-scores of C3NET are higher compared to the other two GRNI algorithms.

**Table 1:** Summary of various F-score statistics

		C3NET	ARACNE	MRNET
Ecoli_100_	max	0.5803	0.4985	0.5648
	min	0.3879	0.2109	0.3109
	median	0.4951	0.3601	0.4890
	mean	0.4890	0.3578	0.4781

Yeast_20_	max	0.3786	0.3543	0.3505
	min	0.192	0.2231	0.2615
	median	0.2810	0.2869	0.3103
	mean	0.2805	0.2825	0.3122

DAG	max	0.7752	0.7684	0.7441
	min	0.6206	0.6041	0.5917
	median	0.7272	0.6947	0.6904
	mean	0.7242	0.6945	0.6895

RND	max	0.4930	0.5102	0.4385
	min	0.3333	0.3056	0.3183
	median	0.4139	0.4017	0.3709
	mean	0.4140	0.4036	0.3705

SF	max	0.3620	0.3037	0.3851
	min	0.2774	0.1946	0.2633
	median	0.3198	0.2521	0.3326
	mean	0.3200	0.2516	0.3301

Summary of F-scores (max, min, mean and median) for C3NET, ARACNE and MRNET for various different network types. The sample size is 1000 for all networks except *E. coli *and *yeast*, where sample size was 100 and 20, as indicated.

**Figure 5:**
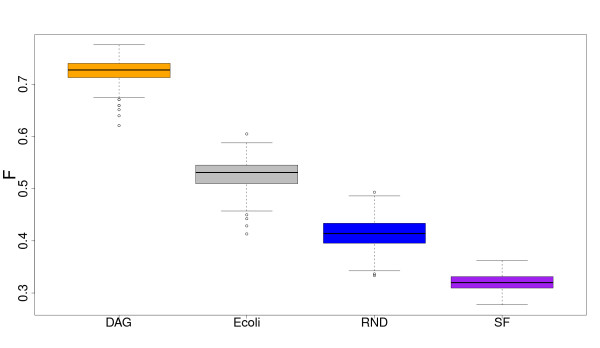
**Performance of C3NET with respect to different network types**. Each network consists of 100 genes. Sample size is 1000 for each of the 300 data sets generated for each network type. DAG: Directed acyclic graph. E coli: Subnetwork from the transcriptional regulatory network of *E. coli*. RND: Random network. SF: Scale-free network.

In order to clearly illustrate the inference performance of C3NET in dependence on the different network types, we plot in Figure 5 a summary of the corresponding F-scores. This figure suggest that C3NET obtains its best inference performance for DAG networks, with a significant margin to all other network types. The second best performance is obtained for the (real) biological network of *E. coli*, with an about 0.2 lower F-score. Then, the RND network follows and finally the SF network, with a median F-score that is slightly larger than 0.3. This figure demonstrates also that C3NET behaves differently for all four network types. This means that none of the three synthetic networks could serve as a 'good model' for the transcriptional regulatory network of *E. coli*. Interestingly, the RND network is closest with respect to the F-scores obtained for the subnetwork of *E. coli*.

It is important to note that the results presented in this section were obtained by using the optimal threshold values (*I*_0_) for all algorithms (C3NET, ARACNE and MRNET). This threshold is used in the first step in each of the algorithms. Apparently, the optimal cut-off value can only be obtained in studies where the (true) reference network is known. With respect to the obtained results, this implies that our results represent upper bounds which cannot be exceeded by any method trying to estimate this cut-off value from the data. For instance, performing a resampling of the data in order to estimate the cut-off value from the sampling distribution of the null hypothesis for each algorithm could not result in better results but leads only to similar results at best.

### Local Network-based performance metrics

In this section, we analyze the inference performances of C3NET locally, investigating various network types by using local network-based performance metrics. The local network-based measures used in this section have been introduced in [36,37].

**Figure 6:**
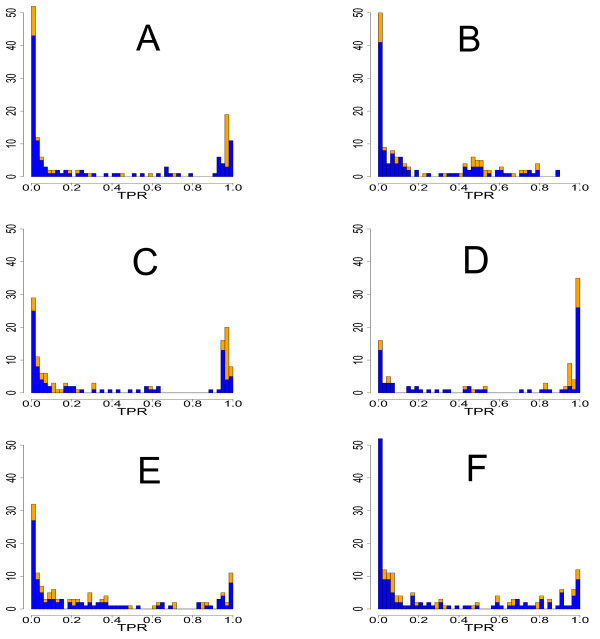
**Histogram of true positive rates for edges in the network for C3NET**. A: Subnetwork of *yeast *(sample size 200). B: Subnetwork of yeast (sample size 20). C: Subnetwork of *E. coli *(sample size 1000). D: DAG-like network (sample size 1000). E: Random network (sample size 1000). F. Scale-free network (sample size 1000). Blue indicates the contribution from activator and orange from repressor edges.

The first property we study is the influence of activator (positive effect) and repressor (negative effect) edges on the inferability of the network. This property of an edge is given by the dynamical equations we used to simulate expression data. If a gene has a positive coupling to a second gene, this edge is called activator, if the coupling is negative, this edge is termed repressor. Overall, this means we study the inference performance of C3NET with respect to a binary edge type because an edge is either an activator or an repressor but cannot be both. In Figure 6 we show histograms for various sample sizes and networks, to visualize the effect of activator edges (blue) and repressor edges (orange) on the true positive rate (TPR) of individual edges. The TPR of an edge is the fraction of the number of times a specific edge is correctly inferred, divided by the size of the ensemble (300). In order to assess the results in Figure 6 quantitatively, we apply a two-sample Kolmogorov-Smirnov test [50] for testing for differences in the cumulative distribution function (CDF) of activator and repressor edges. Coarsely speaking, this test reveals whether the CDFs of the two edge types show heterogeneous behavior or not, when C3NET is used as inference algorithm.

Figure 6 A shows the results for a subnetwork of *yeast *that consists of 100 genes. The sample size used for these simulations was 200. Figure 6B is obtained for the same network, however, for a sample size of 20. This allows to study the influence of the sample size on the performance of C3NET. Figure 6C is obtained from a subnetwork of *E. coli*, Figure 6D is from a DAG, Figure 6E is from a random network and Figure 6F is obtained from a scale-free network. All these networks contain 100 genes and their data set has a sample size of 1000. For the subnetwork of *yeast *with sample size 200, a two-sample Kolmogorov-Smirnov test gives a p-value of 0.002785. For the same network, but a sample size of 20, we obtain a p-value of 0.08638. This result suggests that, for a significance level of *α *= 0.01, the edge type has a systematic effect on the inferability of C3NET, if the sample size is large. This can be confirmed visually from the histogram shown in Figure 6A. Here the repressor edges (orange) have a notably higher TPR and are, thus, easier to infer by C3NET. Overall, this means that C3NET is sensitive to the used sample size. For a sample size of 20, the p-value appears not significant suggesting that there is no heterogeneous behavior in the CDFs and, thus, we cannot conclude that one of the edge types is better inferable than the other. For the *E. coli *network, a Kolmogorov-Smirnov test results in a p-value of 0.002112. The result suggests that, for a significance level of *α *= 0.01, the edge type has a systematic effect on the inference of the *E. coli *network using C3NET. If we look at the histogram for this network in Figure 6, we qualitatively observe that the repressor edges (orange) again seem to have higher TPRs and, thus, are easier to infer with C3NET. For the three synthetic networks a two-sample Kolmogorov-Smirnov test results in the following p-values: 0.02942, 0.1238, 0.2387 for the DAG, RND and SF network, respectively. These results suggest that for neither network the edge type has a systematic effect on its inference (for a significance level of *α *= 0.01), although, the DAG network shows a certain tendency toward it. However, considering the large sample size used for these results it appears sensible for not considering this p-value as significant. Interestingly, all these results point to a crucial difference between the results for synthetic and real biological networks. Overall our results suggest that C3NET's inference performance is affected by the edge type only for real biological networks for large sample sizes. The fact that C3NET is effected for large, but not small samples sizes means that this dependence appears not to be spurious. (If it would be reversed it would indicate a systematic bias introduced by the sample size because the chosen value of the lower sample size is somewhat arbitrary whereas the large sample size is an approximation of the asymptotic behavior of C3NET.) For the *yeast *and *E. coli *network the repressor edges seem to be better inferable than activator edges. For all synthetic networks the edge type has no effect on the inference performances.

**Figure 7:**
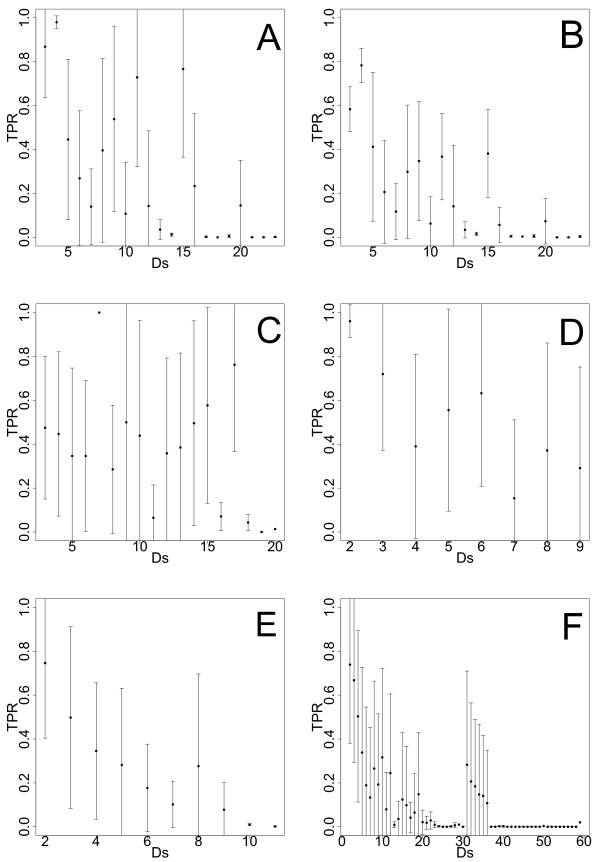
**TPR in dependence on the gene-degree specific measure *D_s_***. **A**: *Yeast *(sample size 200). B: *Yeast *(sample size 20). C: *E. coli *(sample size 1000). D: DAG network (sample size 1000). E: Random network (sample size 1000). F: Scale-free network (sample size 1000).

The second network-based measure we use allows to detect a possible effects of the local network structure surrounding an edge, on its inferability. More precisely, we de ne the metric, *Ds_ij _*, as the sum of the out-degree of node *i *plus the in-degree of node *j *[37]. Here the edge between *i *and *j *refers to a directed connection. The effect of *Ds_ij _*on the mean TPR  of edges is in the following used to asses the performance of C3NET. For this analysis we use the same data sets and the networks we used in Figure 6. Figure 7 illustrates the functional relationship between the mean TPR  and *Ds *for all networks. Here the order of the network type is the same as in Figure 6. In order to quantitatively investigate whether there is a systematic effect of *D_s _*on  we apply an one-factor ANOVA test to test for equal means of the  for each network type. The six ANOVA tests give p-values of 7.35 × 10^-6^, 0.01191, 0.02172, 5.21 × 10^-6^, 5.39 × 10^-7^, 6.63 × 10^-6 ^respectively for the subnetwork of *yeast *with sample size 20, the subnetwork of *yeast *with sample size 200, the subnetwork of *E. coli*, DAG-like, RND and SF networks all with sample size 1000. For a significance level of *α *= 0.01, all three synthetic networks and the *yeast *subnetwork with sample size 20 show a heterogeneous behavior of the  with respect to *Ds *using C3NET. This suggests that the inference performance of C3NET with respect to the  is systematically affected by the values of *Ds *and, hence, the local network structure directly surrounding an edge, for those four networks. This can also be observed from the figures, which leads to the conclusion that as *D_s _*gets larger the values of the  decrease. On the other hand, our results do not suggest a systematic dependence of  on *Ds *for the subnetwork of *yeast *(sample size 200) and the subnetwork of *E. coli *(sample size 1000). Again, given the large sample size for these cases, but also the large margin to the p-values of the three synthetic networks, suggests, that there is a significant difference between the results for the biological and synthetic networks. Therefore, we conclude that, in general, C3NET's inference performance is for the three synthetic networks significantly influenced by the values of *Ds*, but only moderately effected for biological networks.

**Figure 8:**
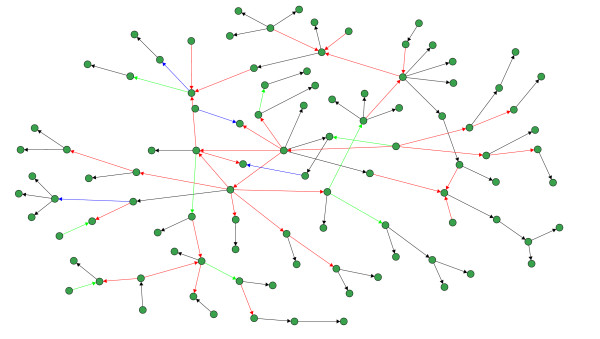
**Directed network motifs with three genes**. 1) chain, 2) collider, 3) fork, 4) triangle.

Next, we asses the performance of C3NET with respect to basic motif types consisting of three genes. For this purpose, we use a motif metric also used in [36,37]. For each motif type we calculate the *true reconstruction rate *which is the sum of the true positive rates (TPR) for existing and true negative rates for non-existing edges in each motif type. The details of this local network-based measures can be found in [36,37]. In Figure 8, we show the four motif types used for our analysis. These are directed motifs resulting from the interactions of three genes. The results for C3NET of our numerical analysis are presented in Table 2. In this table,  represents the mean true reconstruction rate, #*m *represents the number of motifs found in a network and  is the standard deviation of .

**Table 2:** Summary of the statistics for the inferability of motifs

	measure/motif type	1	2	3	4
Ecoli	#*m*	33	59	549	7
		0.518	0.378	0.657	0.232
		0.141	0.055	0.255	0.126

Yeast	#*m*	40	171	446	10
		0.592	0.360	0.592	0.190
		0.165	0.052	0.236	0.150

DAG	#*m*	109	16	94	2
		0.637	0.416	0.675	0.233
		0.178	0.087	0.217	0.204

RND	#*m*	161	110	89	4
		0.5062	0.4229	0.5549	0.0836
		0.143	0.083	0.163	0.045

SF	#*m*	1363	792	940	258
		0.4160	0.3681	0.4893	0.0592
		0.119	0.072	0.161	0.099

Summary of motif statistics for all considered networks. #*m *is the number of motifs,  is the mean true reconstruction rate for a motif and *σ *(*p*) is its standard deviation. The sample size is for all cases 1000 except the *Yeast *network that has sample size 200. For all simulations the size of the ensemble was 300.

From Table 2 we observe that C3NET consistently infers motifs of type 3 better than the remaining motifs, for all network types, with respect to the mean true reconstruction rate. Then motif type 1 allows the second best inference. It is worth mentioning that it has only slightly lower  values and, hence, has a similar good inferability as motifs of type 3. Motifs of type 2 rank third, however, there is already a significant gap to motifs of type 1 and 3. Motifs of type 4 cause the biggest difficulties which can be seen from their low  values. However, in most networks this motif type is only observed a few times (low #*m *values) which may result in unreliable estimates. Only in the SF network motifs of type 4 can be found multiply. For this network, the results for  clearly indicate that C3NET can hardly infer motifs of this type.

**Figure 9:**
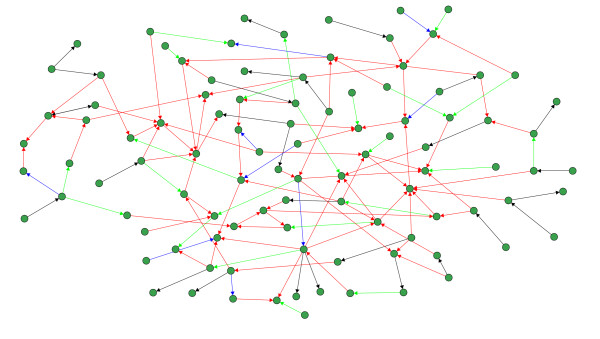
**Inferability of a DAG-like network consisting of 100 genes (sample size 1000)**. Black edges, 1 ≥  > 0.75, blue edges, 0.75 ≥  > 0.5, green edges, 0.5 ≥  > 0.25, and red edges, 0.25 ≥  ≥ 0.0.

**Figure 10:**
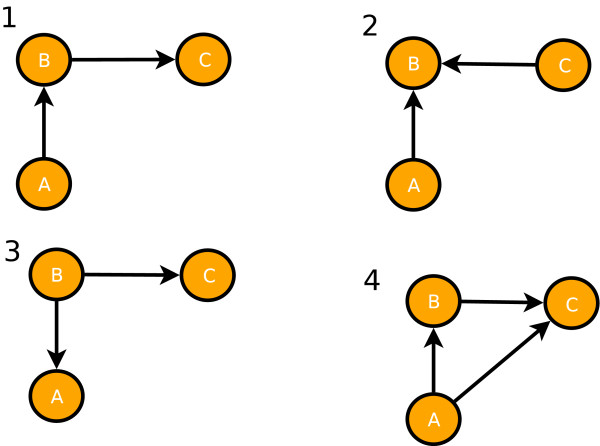
**Inferability of a random network consisting of 100 genes (sample size 1000)**. Black edges, 1 ≥  > 0.75, blue edges, 0.75 ≥  > 0.5, green edges, 0.5 ≥  > 0.25, and red edges, 0.25 ≥  ≥ 0.0.

The last local network-based measure we use in our analysis assesses the inferability of every single edge in a network and, thus, provides the nest resolution of any error measure since an edge is the most basic component of a network [36,37]. In the following, we evaluate the mean TPR of each edge. For reasons of simplicity, we divide the values of TPR into four categories and visualize the edges in the networks correspondingly. Specifically, we are using the following color code for Figure 9 and Figure 10: Black edges, 1 ≥  > 0.75, blue edges, 0.75 ≥  > 0.5, green edges, 0.5 ≥  > 0.25, and red edges, 0.25 ≥  ≥ 0.0. Visualizations of our results for a DAG and a RND network are shown in Figure 9 and Figure 10. The sample size for these simulations was 1000. From these networks we observe that C3NET infers all leaf edges, because all leaf edges in all networks are colored black (for SF we obtain similar results - not shown). Here a leaf edge is defined as an edge that connects to a leaf node, which has only one incoming edge and no outgoing edges. The results for the motifs in Table 2 are also supportive for this finding regarding the inferability of leaf edges (Figure 9 to Figure 10), because a motif of type 3 can be formed by two leaf edges. This observation allows us to hypothesize that C3NET can easily infer the leaf edges for all the considered network types. It can be further observed from the red edges that C3NET has difficulties in inferring the cross connected edges that are 'deeper' inside the network.

### Cut-off effects

**Figure 11:**
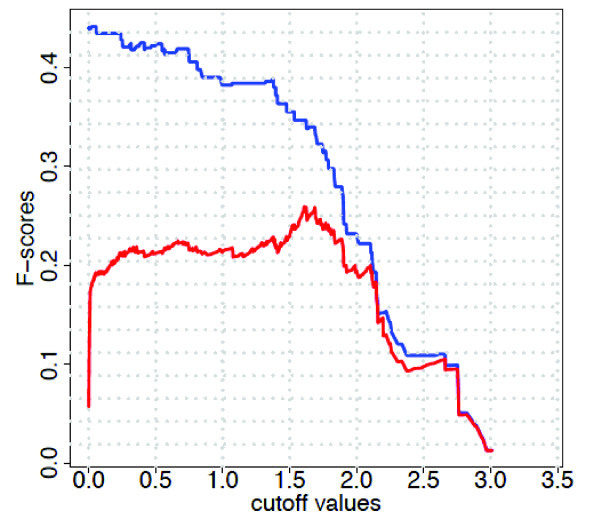
**F-score vs MI cut-off**. The blue line is for C3NET and the red for RN (relevance network).

Finally, we investigate the influence and the sensitivity of the cut-off value *I*_0 _on C3NET. This cut-off value is used in the first step of C3NET in order to eliminate non-significant edges, as described in the methods section. Practically, this cut-off value needs to be estimated by resampling the data and selecting a significance level *α*. However, if the estimated cut-off value deviates from the *optimal *cut-off value, it may lead to a decrease in the performance of the inference algorithm that uses this cut-off value. In order to analyze the influence of *I*_0 _on C3NET we vary its value systematically within a wide range and observe the inference performance of C3NET for each of these values by calculating F-scores. Since the first step of C3NET is equivalent to RN, we also illustrate its behavior in Figure 11 to have a comparison. For the results shown in this figure, we used the subnetwork of *yeast *and one data set with sample size 200 (result for other data sets and network types look similar, not shown). From this figure we observe at least two things. First, there is a large margin between the F-scores for C3NET and RN. Hence, any cut-off value below ~1.75 leads to better results which makes the finding of the optimal cut-off value for C3NET less important. Second, the optimal cut-off value for C3NET assumes usually quite low values, but any value up to about ~1.0 results in F-scores that are only a couple of percentages worse than for the optimal value. This indicates the robustness of C3NET and the existence of a at plateau around the optimal cut-off value with respect to F-scores.

## Conclusions

In this study we investigated the influence of the structure of gene networks on their inference by using C3NET. Our analysis using a global performance metric demonstrated that C3NET provides consistently superior or at least competitive results compared to other inference algorithms widely used [27,30]. This result holds robustly for different biological and synthetic networks. Other inference algorithms show a more sensitive behavior in dependence on the used network type. Interestingly, the global performance metric revealed that the inference performance of C3NET is best for a DAG network followed by a subnetwork of the transcriptional regulatory network of *E. coli*, whereas the SF network received the lowest F-scores. This points to a crucial difference of the latter two network types. As discussed brie y in the 'Methods' section, one network property, in this case the scale-free behavior of the degrees, is not sufficient to determine a network. Hence, the assumption that a SF network may be a good model of real transcription regulatory networks is ill-posed. Our results suggest that the algorithm used by GeNGe [43] to generate SF networks should be revised in order to produce scale-free networks that are more close to biological transcriptional regulatory networks. A general discussion of the point follows below.

From a complementing analysis, using local network-based performance metrics, we found the following. First, C3NET is differently affected by repressor and activator edges for the biological networks only. For these networks, repressor edges are in general easier to infer than activator edges. This corresponds with results obtained for ARCNE, CLR, MRNET and RN which showed also a significant behavior [37]. This point discloses a general concern many GRNI algorithms seem to suffer from. As a potential reason for this problem we speculate that activating effects may be 'additive' whereas repressor edge are more canalizing [51]. This would imply that repressor edges act more *decisive *than activators which may be involved in the logical control of other genes, as a collective. Second, the inference performance of C3NET is affected by the sum of the in-degree and out-degree of edges (*D_s_*), but only for synthetic networks. This is in contrast to the GRNI algorithms ARACNE and CLR which showed a significant dependency on *D_s _*[37]. Due to the fact that significant results point to a bias in the inference abilities of a GRNI algorithm, C3NET maybe preferred over these methods in order to circumvent potential problems. Third, we showed that the inference performance of C3NET depends for all studied networks on the type of a motif. This observation is also supported by the visualization of the networks which indicates that leaf edges have in general a very high inferability. Due to the fact that motifs of type 3 can be formed by two leaf edges, in contrast to all other motif types, it is plausible that this motif type can be inferred best. Taken together, the information gathered from these complex dependencies of C3NET on the conditions of the underlying gene regulatory network are an important source of information for theoretical but also practical reasons. First, the results of our analysis allow to address specific aspects of the inference algorithm in order to refine C3NET. It is immediately clear that pinpointing a problem is the first step of revising any method. For this reason, our results can also be seen as an exploratory analysis in this context. Second, for potential users of our algorithm the obtained information may be helpful in selecting among various available algorithms for the inference of gene regulatory networks. This practical point is especially important because, as demonstrated by our analysis, the behavior of an inference algorithm is not straight forward to predict for given conditions but there are subtitle differences among the available methods. Depending on the intended application purpose and the characteristics of the domain, especially for biomedical data, these differences may be weighted differently by different users. Our comprehensive analysis offers a rich source of information for potential users in order to make a con dent selection. Third, in order to make it easier for biologists to use C3NET we provide a freely available and user-friendly R implementation.

In addition to the above results, our investigations revealed another interesting point, which is, in fact, not directly related to the inference of regulatory networks. Instead, this point relates to the differences between real biological and synthetic networks as observed in the significantly different behavior of C3NET. More precisely, the comparison of F-scores (see Figure 5) but also *D_s _*(see Figure 7 and the results from ANOVA tests) showed that there is a clear discrepancy between these two types of networks in the way that none of the studied synthetic networks could serve as a 'good model' of real biological networks. Or in other words, the synthetic networks seem to lack important properties resulting in discriminatory features that reflect in the performance of C3NET. Turned differently, as assessment if a synthetic network is capable of mimicking, e.g., a transcriptional regulatory network one could compare the inferential characteristics of a GRNI algorithm in order to judge complementing features that are based on graph-theoretical properties [52-55].

A problem that is of eminent importance for the practical application of any inference algorithm is the estimation of the threshold parameter *I*_0_. Due to the fact that all algorithms use nonparametric tests this requires the appropriate randomization of the data. Computationally, this possess a challenge for a comprehensive analysis.

On a more general note, the large-scale inference of statistical entities [56,57], for instance of gene regulatory networks, possesses many difficulties and we are facing unprecedented problems. However, due to an intensified effort of the community [58,59] it can be expected that we are entering an era that will lead to dramatic changes and further developments in the computational but especially statistical methodologies involved in this endeavor. Such developments are necessary, in order to deal with high-throughput data reliably allowing to connect basic biological and medical research programs [60].

## Competing interests

The authors declare that they have no competing interests.

## Authors' contributions

GA and FES designed the method, performed the analysis and interpreted the results. FES conceived and coordinated the study. GA and FES wrote the manuscript. All authors read and approved the final manuscript.

## Reviewers' comments

### Reviewer's report 1

**Lev Klebanov, Department of Probability and Statistics, Charles University, Czech Republic**.

This is an interesting paper analyzing the inferential characteristics of a new regulatory network inference algorithm, C3NET with respect to different network types of gene networks. Specifically, authors study the influence of various network structures, two biological as well as three synthetic ones, using global and local performance metrics. They use four different types of local-network based measures to assess the performance of C3NET. Authors mentioned that there may be parts or subnetworks, e.g., motifs or modules, of the overall network that may be significantly better to infer than others. In order to identify such substructures local network-based measures allow to zoom in these structural regions, and therefore they provide an introduction to the usage of c3net, a R implementation of C3NET. The software package c3net is available from the web site https://r-forge.r-project.org/projects/c3net. The usage of the function and its default parameters are as follows: c3net(dataset, alpha = 0.01, methodstep1 = "cutoff", cutoff MI = 0, MTCmethod="BH", itnum = 5,network = FALSE). Here dataset is the data set and alpha is a user defined significance level. For the parameter methodstep1 one can assign three different options, "cutoff", "MTC", "justp", in order to choose a procedure to eliminate nonsignificant edges. Let us note that available options of different MTC methods are "BH", "bonferroni", "BY", "hochberg", "holm", "hommel". Also, "itnum" needs to be set to specify the number of iterations to obtain a null distribution and alpha the statistical significance level. If methodstep1 = "justp" then only alpha and itnum need to be set. Authors generated simulated expression data, including biological noise, by using SynTReN. As it was mentioned above, the manuscript seems to be interesting, and contains a lot of new results. However, I have some comments (see below) concerning the use of proposed software.

Comments:

1. As it was mentioned above, some parameters and/or options like "alpha","BH", "BY" and so on, has to be defined. Unfortunately, the authors did not mention how sensible is the model to the choice of the parameters and options. For example, how will it change if one will use *α *= 0.05 instead of *α *= 0.01? In reality, one does not know what level alpha has to be used. If the result will changed dramatically, it will show that the system cannot be used. In contrary, the system has to be sensible to large changes of significance level.

2. The same question may be addressed to the option in MTC. For example, it is not clear, what should be difference between "bonferroni" and "hochberg". However, the difference between "bonferroni" and "holm" should not be dramatical (from my view).

3. As it was mentioned above, the authors simulate expression data, including biological noise. I do not understand, what distribution have corresponding random variables. If it is Gaussian distribution, than the use of Pearson correlation coefficient is absolutely correct, and one does not need to use mutual information (MI) (for Gaussian distribution there are no nonlinear effects). Because the authors use MI, they, probably, have non-Gaussian distribution for either expression data or for biological noise. What is this distribution, and what are the reasons for its use.

#### Author's response

Answer to 1: The reviewer addresses a very important point. Due to the fact that the inference of regulatory networks is a multi-step procedure rather than a monolithic method we focused in this paper on the influence of the underlying network structure on its inference. That means we did not attempt to address all problems explicitly. In our experience, the influence of the significance level needs to be discussed in a method-specific manner. The reason for this is that each method employs a different philosophy in applying the hypotheses tests. With respect to C3NET the exact value of *α *has no large influence on its performance as long as the chosen significance level is reasonable with respect to the noise in the data. Specifically, C3NET allows each gene to add *at most *one edge to another gene. This edge has also the maximum mutual information value between this gene and all other genes in the study and, hence, this edge has also the lowest p-value of all these edges. Numerically, we found that C3NET behaves very robustly with respect to small changes in *α*. However, we agree with the author that this needs to be studied comprehensively.

Answer to 2: The problem of multiple testing corrections (MTC) is in the literature of GRN not very well studied and deserves much more attention. Due to the correlation among the test statistics (because otherwise no network could be inferred) only MTCs taking this into account should be recommended. However, we think that none of the available methods is optimally designed for this type of problem, demanding a methodological extension.

Answer to 3: For the generation of simulated expression data, we employed SynTReN [47]. In [47] it is described that a lognormal distribution is used to model noise, justified by experimental findings in [48]. Currently, there are many algorithms available for generating simulated expression data. The reason for using SynTReN instead of another algorithm is that it is well known in the community. We added a description of this to the section 'Simulation methodology'.

We are very grateful for your comments.

### Reviewer's report 2

Joel Bader, Johns Hopkins University, School of Medicine, USA.

General comments: This manuscript presents a comparison of gene regulatory network inference methods on expression data generated by simulation from network frameworks subsampled from existing databases and generated by different random network models. The comparisons are flawed for a number of reasons. First, the results are probably over-optimistic. The networks are quite small, with 100 vertices, making the problem much easier than real networks. The amount of noise in the simulated data is not described explicitly. Important parameters are selected using the true data to guide parameter choice. For all these reasons, the absolute performance is likely to be better than that when applied to real data, and the ordering of algorithms might change for real data. Furthermore, the structure of the network itself might favor one algorithm over another. The C3NET algorithm restricts its final prediction to a minimal spanning tree (MSTs). If the true network has edges that are not part of a minimal spanning tree, these edges will be necessarily missed by C3NET. Networks that are closer to MSTs will be easier for C3NET and, other aspects being similar, harder for algorithms that generate more general network topologies. Therefore the sparsity of the networks simulated could affect the performance. For a broader comparison, there have been DREAM challenges that pose the problem of network inference. It would be better to apply C3NET and the other algorithms to this public data for a better calibration with other algorithms. Finally, for readability, the authors should make the manuscript and the figure captions more self-contained. The algorithms should be presented as part of the methods, and the differences between algorithms should be made clear.

Detailed review: This manuscript investigates the performance of C3NET, a method developed to infer the structure of gene regulatory networks (GRNs) from gene expression data. C3NET is compared with ARACNE, another GRN inference method. Data sets are generated from network models designed to mimic expression data from GRNs with 100 genes. The methods section states that "In the first step of C3NET, aiming at the elimination of nonsignificant edges, we used the optimal cut-off value, which is the threshold (I0) that maximizes the F-score for each data set with respect to the true underlying network structure [36,37]." This would seem to give an unfair advantage to C3NET, since in any application to real data the true network will be unknown. It is not clear how the parameters are set for other methods. On p. 7 the authors state "The DPI tolerance parameter of ARACNE, when used for comparison purposes, was chosen as 0.1 [30]," but later they state "the results presented in this section were obtained by using the optimal threshold values (I0) for all algorithms (C3NET, ARACNE and MRNET)." These statements seem inconsistent. This also creates questions about how the results for synthetic data would look within tuning. The authors must describe how to set the parameters appropriately without knowledge of the true network structure. There have been several assessments of GRN inference through the DREAM challenge, and ideally it would be worthwhile to apply to some of these published data sets also. The manuscript should be modified to make the methods and results more self-contained. While some references to technical details are acceptable, it is too much to ask a reader to refer to previous papers for essential information such as the method itself (which can be compactly specified), for performance metrics, etc. The discussion of the performance for different edge types, p. 9, is a bit confusing because two concepts are being explored: (1) the recall or true positive rate of activator vs. repressor edges; (2) the p-value for a test that compares TPR for activator vs. TPR of repressor. The authors show that, with sufficient samples, the p-value is significant. But it is also important to provide the estimates for the TPR for the two types of edges. Since edges in random networks occur with probability proportional to the product of in-degree and out-degree, the degree product (or its log) might be a better covariate to explore than the degree sum (p. 9). Again, estimates of the size of the effect should accompany calculations of p-values. For the inference of leaf nodes, p. 11, it is important to know whether the simulated network has more leaf nodes than the larger networks they were sampled from. This would affect performance with real data. The figure captions should be revised to permit figures to be understood without referring to the main text.

#### Author's response

We agree with the reviewer that it is very important to apply the same conditions for all methods. This point is explicitly emphasized in section 'Global performance metric' in the last paragraph. For this reason, we used for all studied algorithms their optimal parameter values in order to provide a fair comparison. We improved this explanation in the main text. We used 100 genes in our networks in order to reduce the computational complexity of the problem. This way we were able to study thousands of different data sets, instead of a single one as is the case for biological data, gaining insights into the population behavior of the studied inference methods. Due to the fact that we are studying an inferential problem this approach has advantages from a statistical point of view. The reviewer is completely right in stating that the structure of the network influences their inference. That is why we decided to make this the major topic of this paper because this fact has not been studied before systematically.

As explained above in our response to the comments made by reviewer 1, the inference of a GRN is a multi-step procedure. For reasons of clarity we did not aim to address all problems that this topic offers, instead, we focused on a few questions. The reviewer is entirely right pointing out that the randomization of the data, in order to obtain estimates for the threshold *I*_0_, is of eminent importance. Due to the fact that this is an intricate statistical problem our ensemble approach seems to be well suited to study this problem in more detail from the population perspective. From a computational perspective this possess a considerable problem, though, because one would need to repeat our study 10000 times (typical number of resampling steps). Hence, for a comprehensive analysis this is quite challenging. This is certainly a disadvantage of the usage of nonparametric methods. We added a discussion of this point to the section 'conclusions'.

We improved the description of our results for activator and repressor edges. The point is that from an abstract point of view we study the inference performance of different classes of edges. Each such class is defined by certain properties, e.g., the degree of the edge enclosing genes, or the type of an edge (activator or repressor). For this reason each edge is not only studied once but appears in each different class. The estimates for the TPR for activator and repressor edges is shown in Figure 6. We revised the text in order to clarify this.

The reviewer's suggestion to use the logarithm of the product of the degrees of genes, instead, of their sum as used in this paper, is a good idea. We tried several different measures, including a similar one as the suggested, and found essentially similar results as reported in the results section. However, we did not study this comprehensively.

The used network structures for *E. coli *and *yeast *were obtained by randomly sampling the transcriptional regulatory networks of these organisms, employing SynTReN. That means these networks are representatives of the larger networks, within statistical variations. The total number of leaf nodes does actually not influence the finding that the corresponding leaf edges, statistically, are better to infer than other edges. However, we agree that the network structure has a profound influence on the inferability. All figure captions have been revised in order to make them more understandable.

The methods section has been extended including more detailed explanations of the used inference algorithms.

We are very grateful for your comments.

### Reviewer's report 3

Yuriy Gusev, Lombardi Cancer Center, Georgetown University, USA.

This reviewer provided no comments for publication.

#### Author's response

We are very grateful for your comments.
